# LINKS BETWEEN POSITIVE AFFECT AND DISENGAGEMENT FROM NEGATIVE STIMULI IN YOUNGER AND OLDER ADULTS

**DOI:** 10.1093/geroni/igz038.1124

**Published:** 2019-11-08

**Authors:** Kathryn L Ossenfort, Derek M Isaacowitz

**Affiliations:** Northeastern University, Boston, Massachusetts, United States

## Abstract

Older adults attend to more positive than negative content compared to younger adults; this “age-related positivity” effect is often thought of as a way older adults may be regulating their moods. However, attentional disengagement abilities decline with age, which may make positive looking more challenging for older adults in some cases. To evaluate links between early attentional processes and affect, 48 younger adult and 49 older adult participants reported levels of positive and negative affect on the Positive and Negative Affect Scale (PANAS) and completed a spatial cueing task evaluating attentional orienting and disengagement from emotional stimuli. Participants were tasked with responding to the location of a spatial target after seeing a cue (emotional image) that either appeared on the same (orienting) or opposite (disengagement) side of the screen. Multilevel modeling analyses were conducted using age and self-reported affect from the PANAS as predictors at level-2, and trial characteristics as predictors at level-1. Positive affect (PA) was unrelated to task performance for younger adults. Older adults reporting higher PA responded more slowly overall, and higher PA scores predicted similar response times to positive and negative stimuli on both trial types. Older adults reporting lower PA oriented attention more quickly to positive stimuli, but took longer to disengage from negative. These results suggest that there may be a relationship between the ability to flexibly disengage from negative content and PA for older, but not younger adults, and also highlight the importance of teasing apart specific attentional processes when evaluating positivity effects.

